# Facile Preparation of Durable and Eco-Friendly Superhydrophobic Filter with Self-Healing Ability for Efficient Oil/Water Separation

**DOI:** 10.3390/membranes13090793

**Published:** 2023-09-13

**Authors:** Wei Xin Voo, Woon Chan Chong, Hui Chieh Teoh, Woei Jye Lau, Yi Jing Chan, Ying Tao Chung

**Affiliations:** 1 Lee Kong Chian Faculty of Engineering and Science, Universiti Tunku Abdul Rahman, Sungai Long Campus, Jalan Sungai Long, Cheras, Kajang 43000, Selangor, Malaysia; wgwenz@1utar.my (W.X.V.); teohhc@utar.edu.my (H.C.T.); 2 Centre for Photonics and Advanced Materials Research, Universiti Tunku Abdul Rahman, Sungai Long Campus, Jalan Sungai Long, Cheras, Kajang 43000, Selangor, Malaysia; 3 Advanced Membrane Technology Research Centre (AMTEC), Faculty of Chemical and Energy Engineering, Universiti Teknologi Malaysia, Johor Bahru 81310, Johor, Malaysia; wjlau@petroleum.utm.my; 4 Department of Chemical and Environmental Engineering, University of Nottingham Malaysia, Jalan Broga, Semenyih 43500, Selangor, Malaysia; yi-jing.chan@nottingham.edu.my; 5 Department of Chemical & Petroleum Engineering, Faculty of Engineering, Technology & Built Environment, UCSI University Kuala Lumpur Campus, Jalan Mandarina Damai 1, Cheras, Kuala Lumpur 56000, Malaysia; chungyt@ucsiuniversity.edu.my

**Keywords:** superhydrophobic cotton filter, myristic acid, oil/water separation, self-healing

## Abstract

The superhydrophobic feature is highly desirable for oil/water separation (OWS) operation to achieve excellent separation efficiency. However, using hazardous materials in fabricating superhydrophobic surfaces is always the main concern. Herein, superhydrophobic filters were prepared via an eco-friendly approach by anchoring silica particles (SiO_2_) onto the cotton fabric surface, followed by surface coating using natural material—myristic acid via a dip coating method. Tetraethyl orthosilicate (TEOS) was used in the synthesis of SiO_2_ particles from the silica sol. In addition, the impact of the drying temperature on the wettability of the superhydrophobic filter was investigated. Moreover, the pristine cotton fabric and as-prepared superhydrophobic cotton filters were characterised based on Fourier-transform infrared (FTIR) spectroscopy, X-ray photoelectron spectroscopy (XPS), scanning electron microscopy coupled with energy dispersive X-ray spectroscopy (SEM-EDX) and contact angle (CA) measurement. The superhydrophobic cotton filter was used to perform OWS using an oil-water mixture containing either chloroform, hexane, toluene, xylene or dichloroethane. The separation efficiency of the OWS using the superhydrophobic filter was as high as 99.9%. Moreover, the superhydrophobic fabric filter also demonstrated excellent durability, chemical stability, self-healing ability and reusability.

## 1. Introduction

Water is one of the essential components for the survival of all living beings. However, the quality of the water is mostly affected due to various human activities. One of the pollution sources is the contamination from oil spillage and the discharge of oily wastewater into the environment, and thus adversely impacts the sustainable development of mankind [1,2,3]. The oil spillage can happen at any moment from upstream to downstream of the oil industry, including exploration, production, transportation, refinery, and storage [4]. Moreover, industries such as food, textile, oil and gas, mining and metal processing are accountable for the production and discharge of oily wastewater [5,6].

Furthermore, oil spills could result in severe economic impacts as spillage is a great waste of natural resources. The notorious Deepwater Horizon oil spill in the Gulf of Mexico was reported to release a massive volume of 100 million barrels to the environment. Nonetheless, only less than 10% of oil was recovered through conventional remediation methods [7]. The company was responsible for the incident and spent $42 billion on the cleanup, compensation, and fines [8].

The readily available treatment and remediation approaches in addressing oil spills and oily wastewater discharge, including oil containment and skimming, centrifugation, gas flotation, coagulation and flocculation, in-situ burning, chemical dispersion, solidification, and bioremediation [4,6,9,10,11] exhibit low selectivity and more importantly are time intensive. Moreover, in an emergency oil spill, the commonly used porous materials often display amphiphilic properties, allowing the material to absorb water and oil during the operation, resulting in inefficient separation [5]. Changing the wetting characteristics, which can be accomplished by altering the surface chemistry and roughness of the membrane or sorbent, can enhance the oil affinity of the porous materials. Superhydrophobicity is the term used to describe the phenomenon of an extreme water-repelling solid surface, where the surface water contact angle (WCA) shall be at least 150° [12]. According to the wetting models developed by Young, Wenzel and Cassie, the superhydrophobic state can be attained by improving the surface roughness through the modification of the hierarchical structure on the surface and/or by altering the surface chemistry to lower the surface energy [12,13,14].

Other than that, artificial superhydrophobic surfaces can be damaged by mechanical damages such as abrasion and scratches during the real OWS process, resulting in the loss of superhydrophobicity of the surface. The changes in superhydrophobicity of the surface would directly affect its efficiency in OWS. Being inspired by the lotus leaves’ natural ability to regenerate superhydrophobicity after being damaged, the incorporation of materials with self-healing properties is expected to mitigate the losses in superhydrophobicity and prolong its service life [15,16]. Besides, the surface must possess good chemical stability to be functional in different environmental conditions.

Researchers attempted many different ways of fabricating superhydrophobic surfaces, either through a single process or a combination of multiple processes. These methods include freeze-drying [17], electrospinning [18], electrodeposition [19], dip-coating [20,21], spray coating [22,23], and hydrothermal methods [24]. Sam et al. [25] prepared a self-healing superhydrophobic cotton fabric through a series of simple dip-coating processes of polydimethylsiloxane (PDMS), ZIF-90 and fluoroalkyl silane (FAS). The prepared cotton fabric exhibited excellent durability and chemical stability, with a high oil/water separation efficiency of 99.5% and a flux of up to 1213 L m^−2^ h^−1^. The prepared cotton fabric also displayed self-healing ability and reusability. Peng et al. [22] developed a superhydrophobic coating with PDMS-grafted silica SiO_2_ and titanium dioxide (TiO_2_). The coating was sprayed onto a stainless steel mesh for OWS, and the separation efficiency was reported to be higher than 98%. However, FAS is potentially hazardous to the environment and human health because of its fluorine content [26]. In addition, the PDMS is too costly and has limited availability in the local market. Therefore, myristic acid is proposed in this study as it is a non-hazardous material and, most importantly, cost-effective. Myristic acid is a naturally occurring fatty acid that can be extracted from nutmeg, coconut and palm skernels [27,28]. These sources are widely available in Malaysia, and therefore, myristic acid is easily accessible within the local market.

In the previous work [29], where the surface roughness of the cotton fabric was enhanced by SiO_2_ particles and further modified with stearic acid to produce the superhydrophobic filter, the optimum TEOS dosage and optimum drying temperature were determined. Herein, myristic acid is employed in the surface modification step instead to evaluate the dependency of the TEOS dosage on the type of materials used in surface modification. Even though using SiO_2_ particles prepared from TEOS to improve the surface roughness is very common, the effect of the dosage of TEOS on the overall hydrophobicity has not been widely reported.

On the other hand, temperature has great significance in enhancing hydrophobicity, but to the best of our knowledge, there was only one relevant study other than our previous work, reporting the effect of heat treatment on the wettability of the superhydrophobic coating made of SiO_2_, TiO_2_ and PDMS [7,30]. The impact of thermal treatment of the surface wettability provided by SiO_2_ and myristic acid (MA) on cotton fabric has not been investigated. The effects of the TEOS dosage and drying temperature towards the filter’s superhydrophobicity were studied in this work. Besides, the fabricated superhydrophobic SiO_2_/MA@cotton filter separated oil from the oil/water mixture. Furthermore, the filter’s durability, self-healing and reusability properties were investigated and reported in this work.

## 2. Materials and Methods

### 2.1. Materials

Myristic acid (MA), tetraethyl orthosilicate (TEOS), ethanol (95%), ammonia solution (25%), acetone, Oil Red O, and chloroform were purchased from Sigma-Aldrich (St. Louis, MO, USA). All chemicals were used as received, and distilled water was used in laboratory experiments. The cotton fabric (160 g/m^2^), adhesive tape and silicon carbide sandpapers (SC-500, SC-1000, and SC-2000) were purchased from local stores.

### 2.2. Preparation of Silica Sol

In this study, silica particles were synthesized from TEOS and coated onto the cotton through a dip coating technique. The silica sols were prepared in reference to an improved Stober method reported by Zhao et al. [31]. The precursor solution was prepared with a concentration of 0.03 mmol/L by mixing 150 mL of ethanol, 1 mL of TEOS and 15 mL of ammonia solution in a conical flask. The solution was stirred continuously at 300 rpm for 6 h at room conditions to obtain a homogenous white suspension. Besides that, another three silica sols were prepared by adding various volumes of TEOS, i.e., 4.5, 9 and 12 mL, into the precursor solution to obtain the concentration of 0.14, 0.27 and 0.36 mmol/L, respectively.

### 2.3. Fabrication of MA/SiO_2_@cotton

The cotton fabric was prepared into the size of 7 cm × 7 cm and rinsed with acetone to remove surface contaminants. The cleaned cotton fabric was dipped into the silica sol with stirring constantly at 100 rpm for 2 h followed by oven drying at 65 °C for 2 h. The oven-dried fabric is denoted as SiO_2_@cotton. In the meantime, a 1 wt% MA solution was obtained by dissolving 1 g of MA in 100 mL ethanol. The SiO_2_@cotton was then immersed into the 1 wt% MA solution at room condition for 2 h with constant stirring at 100 rpm and oven-dried for 2 h at 80 °C. The obtained modified fabric is denoted as MA/SiO_2_@cotton. Three more MA/SiO_2_@cotton filters were fabricated with 0.14 mmol/L of TEOS concentration but at different drying temperatures, i.e., 120, 160 and 200 °C.

### 2.4. Characterisation

The water contact angle (WCA) was employed to evaluate the superhydrophobicity of the fabricated cotton fabric filters, which was measured using a goniometer (OCA15 Pro, Data-Physics, Filderstadt, Germany) with a 2 μL distilled water droplet as the indicator at room condition. Fourier transform infrared spectrometer (FTIR, Nicolet iS10, Thermo Scientific, Waltham, MA, USA) was employed to identify the functional groups on the filter surface. Moreover, the scanning electron microscopy images were captured at 15 kV to study the surface morphology of the cotton fabrics, whereas the energy-dispersive X-ray spectroscopy (SEM-EDX, S-3400N, Hitachi, Tokyo, Japan) was used to perform the elemental analysis of the modified cotton fabric. Besides, the surface composition of the prepared superhydrophobic filter was studied via X-ray photoelectron spectroscopy (XPS, K-Alpha, Thermo Scientific, USA). Besides, atomic force microscopy (AFM, NX10, Park Systems, Santa Clara, CA, USA) was employed to determine the surface roughness of the filter.

### 2.5. Oil/Water Separation (OWS)

A gravity-driven OWS was performed using the superhydrophobic SiO_2_/MA@cotton filter. The oil/water mixture was prepared by mixing 30 mL of oil and 30 mL of distilled water and transferred to the separation device to evaluate the separation flux rate and efficiency. The oils were then dyed red while the water was dyed with methylene blue for better visibility. The separation efficiency (%), *η*, was calculated using Equation (1) to analyse the performance of the filter in OWS. Meanwhile, the separation flux rate (L/m^2^·h), *J*, was determined based on Equation (2).

(1) $$\eta =\frac{M_{2}}{M_{1}}\;\times \;100$$

(2) $$J=\frac{V}{\mathrm{St}}$$

where *M*_1_ and *M*_2_ are the mass of water (g) before and after separation, respectively. *V* is the volume of the collected liquid (L), *S* is the active area (m^2^), and *t* is the recorded period required by the separation (h).

### 2.6. Mechanical and Chemical Durability Test

**Figure 1:**
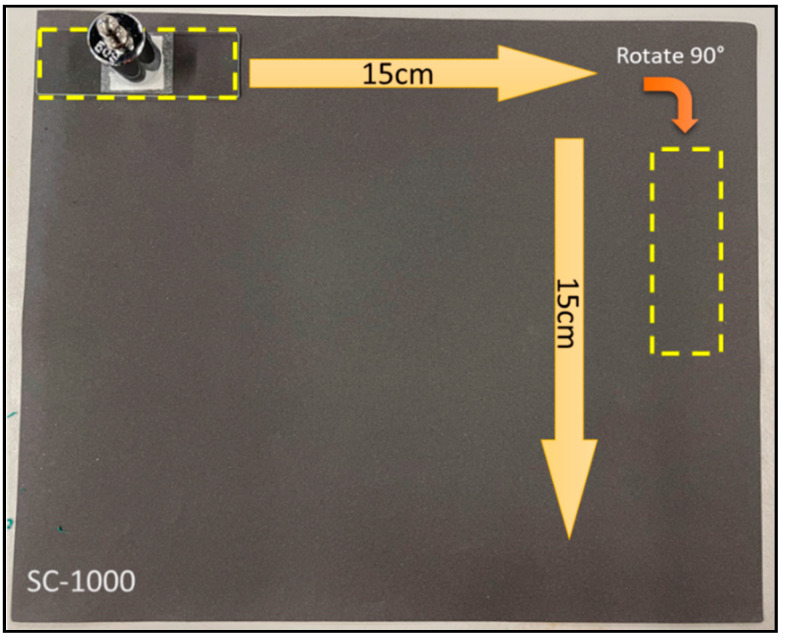
Schematic diagram of sandpaper abrasion test procedure.

In the abrasion test, the filter was adhered onto a glass slide using double-sided tape and then placed on the silicon carbide sandpaper with a constant load of 50 g on top of the glass slide. After that, the glass slide was moved to the right by 15 cm, followed by a 90° turn and moved 15 cm to complete one abrasion cycle [32]. The schematic diagram of the sandpaper abrasion is depicted in Figure 1. The process was repeated 100 cycles, and the WCA was measured after every 20 cycles of the abrasion procedure.

To examine the chemical durability, four solutions of different pH (2, 4, 10 and 12) were prepared using 1 M of HCl and 1 M of NaOH solution to simulate extreme conditions. The MA/SiO_2_@cotton filter was immersed into the prepared pH solutions for 24 h. After that, the filter was withdrawn from the solution, and then deionised water was rinsed and dried in the oven at 80 °C for 20 min, as well as measuring the WCA [33].

## 3. Results and Discussion

### 3.1. Effect of Silica Particles Loading

**Figure 2:**
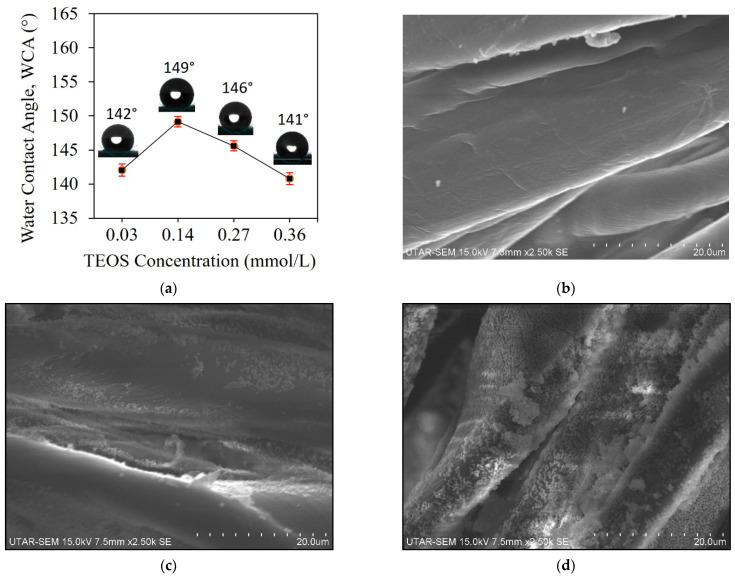

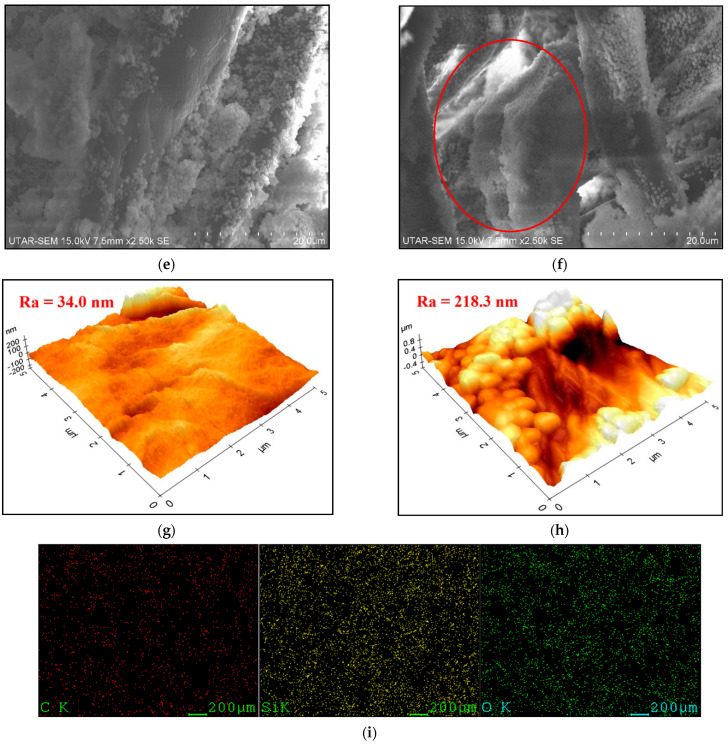
(**a**) WCA of MA/SiO_2_@cotton filter prepared with different TEOS loading. SEM images of the (**b**) pristine cotton fabric and the MA/SiO_2_@cotton filters prepared with various volumes of TEOS at the concentration of (**c**) 0.03, (**d**) 0.14, (**e**) 0.27 and (**f**) 0.36 mmol/L (Note: red circle indicated the large, agglomerated silica particles). 3D AFM surface topography of (**g**) a cotton fabric coated with only MA and the (**h**) MA/SiO_2_@cotton filter. (**i**) Elemental mapping images of C, Si and O elements on the MA/SiO_2_@cotton filter.

In this study, the coating of silica particles is significant in providing surface roughness to enhance the superhydrophobicity of the filter. The WCA measurements of the MA/SiO_2_@cotton filters prepared with different TEOS loading are shown in Figure 2a. The WCA of the filter increased from 142° to 149° when the dosage of TEOS was increased from 0.03 to 0.14 mmol/L. However, the WCA decreased to 146° and 141° when the TEOS concentration was further increased to 0.27 and 0.36 mmol/L, respectively. Similar trends were reported in the work of Vafaei et al. [34], in which the researchers investigated the effect of the bismuth telluride nanoparticles concentration at two different sizes (2.5 and 10.4 nm) on the contact angle of the coated surfaces.

Increasing the chemical loading would result in more particles being formed and anchored on the cotton fibres, which is essential in providing the hierarchical structure of the filter. When the loading continues to increase, aggregation and agglomeration of the silica particles tends to occur, which could form a wide range of particle size. It is a favourable formation at the initial stage as the aggregates and agglomerates provide the rough structure of the cotton fibres [35]. However, as the process extended, more and more silica particles were agglomerated (see Figure 2d), thereby contributing to a lower degree of hydrophobicity due to significant changes in surface energy [36]. The morphology of the pristine cotton fabric and the filters prepared with different TEOS concentrations are depicted in Figure 2 b,c–f, respectively. By observation, the SiO_2_-coated cotton fabrics (see Figure 2c–f) have rougher surfaces with the deposition of the particles compared to the smooth texture of the pristine cotton fabric (see Figure 2b). Besides, this finding coincided with the finding in previous work, which showed that the optimum dosage of TEOS is independent regardless of the material used to modify the surface of the substrate.

On the other hand, the images of 3D AFM surface topography of a cotton fabric modified with only MA and the superhydrophobic MA/SiO_2_@cotton filter, as illustrated in Figure 2g and Figure 2h, respectively, showed that the grafted SiO_2_ particles have greatly enhanced the surface roughness of the cotton fabric. In addition, the images of elemental mapping analysis performed on the MA/SiO_2_@cotton filter prepared with the TEOS concentration of 0.14 mmol/L, as shown in Figure 2i, indicated that the SiO_2_ particles have been successfully coated and evenly distributed on the surface of the filter.

### 3.2. Effect of Drying Temperature

**Figure 3:**
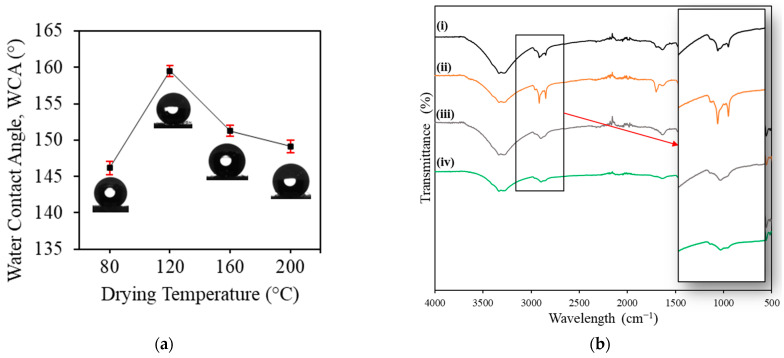
(**a**) WCA of MA/SiO_2_@cotton filters prepared with different drying temperatures; (**b**) FTIR spectra of filters fabricated with different drying temperatures: (i) 80 °C, (ii) 120 °C, (iii) 160 °C and (iv) 200 °C.

Figure 3 depicts the changes in WCA measured for the MA/SiO_2_@cotton filters prepared with 0.14 mmol/L of TEOS concentration under different drying temperatures. It was found that the WCA was significantly increased from 146° to 160° when the drying temperature increased from 80 to 120 °C. The high thermal treatment contributed to the dissociation of hydrogen bonds that prevail particularly on the surface functional groups, for example, the carboxyl group (–COOH), which typically possesses undesirable hydrophilic properties [37,38]. This reduced surface energy and promoted surface hydrophobicity. However, as the drying temperature increased to 160 and 200 °C, the WCA dropped to 151° and 149°, respectively. The FTIR spectra of the filters fabricated under different drying temperatures, as shown in Figure 3b, indicated the changes in the bond intensities of alkyl groups (CH_3_ and CH_2_) at 2800–3000 cm^−1^ could provide the low surface energy for the surface of the filter. The bond intensities of the alkyl groups increased as the drying temperature initially raised, where two characteristic peaks can be observed in the spectrum (i) and even more significant in the spectrum (ii). However, the intensity was then reduced as the temperature further increased. This indicated a decrease in the amount of the alkyl groups present on the filter surface. A similar experiment has been carried out in a previous work where stearic acid was used instead of myristic acid, and the optimum drying temperature for the SiO_2_/stearic acid superhydrophobic filter was found at 200 °C [29]. Hence, it can be deduced that heat treatment can enhance hydrophobicity to a certain extent, as different materials possess different curing and decomposition temperatures.

### 3.3. FTIR and XPS Analysis

**Figure 4:**
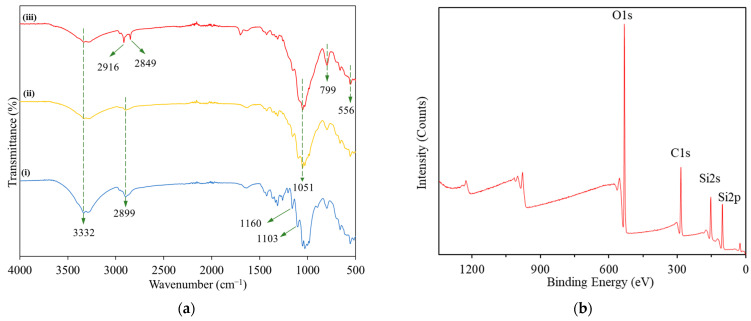

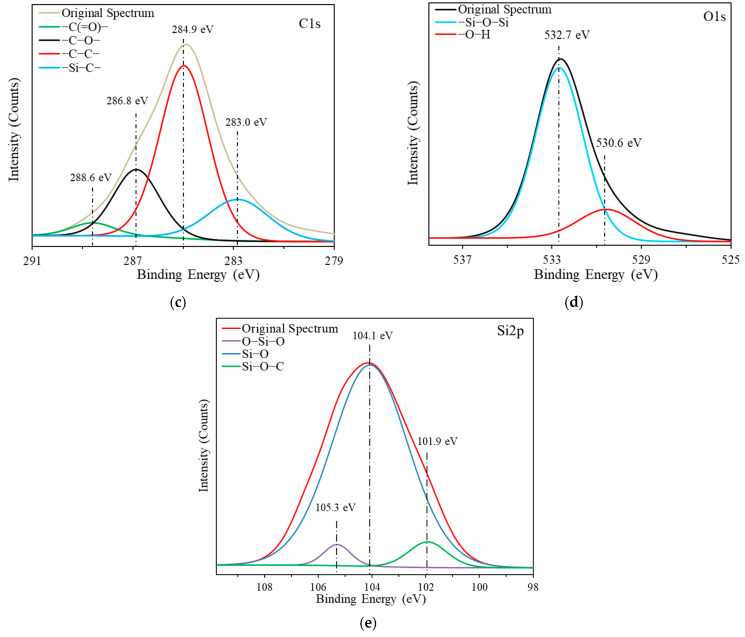
(**a**) FTIR spectra of (i) pristine cotton fabric, (ii) SiO_2_-coated cotton fabric and (iii) MA/SiO_2_@cotton fabric; (**b**) Low-resolution XPS spectrum of MA/SiO_2_@cotton fabric together with high-resolution spectra of (**c**) C 1s, (**d**) O 1s and (**e**) Si 2p.

Figure 4a depicts the FTIR spectra of an uncoated cotton fabric and an MA/SiO_2_@cotton. The peaks that appeared at 1103 cm^−1^ and 1160 cm^−1^ in the spectrum of pristine cotton fabric are assigned to the stretching vibration of the C–O–C and C–O groups [32,39]. Whereas the broad adsorption bands of 2899 cm^−1^ and 3332 cm^−1^ are dedicated to the –CH_2_ and –OH groups stretching vibrations, respectively [40]. These are the characteristic functional groups of a cotton fabric. In the spectrum of MA/SiO_2_@cotton fabric, two distinct absorption peaks presented at 2916 cm^−1^ and 2849 cm^−1^ are respectively dedicated to the symmetrical and asymmetrical stretching of –CH_3_ and –CH_2_ groups, implying the presence of the long alkyl hydrocarbon chain contributed by myristic acid [16]. Besides, the peaks observed at 1051 cm^−1^ and 799 cm^−1^ are the characteristic peaks that indicated the presence of Si–O–Si and Si–CH_3_, respectively [41,42]. Meanwhile, the peak appeared at 556 cm^−1^ also signified the presence of Si–OH in the superhydrophobic cotton fabric [43]. The interaction bonds between the SiO_2_ and MA on the surface of the superhydrophobic filter were signified by the presence of Si–CH_3_ and Si–OH.

To further investigate the surface composition of the filter, XPS analysis was conducted. At the low-resolution XPS spectrum of the superhydrophobic MA/SiO_2_@cotton filter shown in Figure 4b, the peaks corresponding to O 1s (533.1 eV), C 1s (285.1 eV) and Si 2p (104.1 eV) were observed clearly, indicating the presence of these elements on the filter surface [44]. The high-resolution spectra of O 1s, C 1s and Si 2p were further analysed, and the outcomes are depicted in Figure 4c–e. In the C 1s spectrum, there are four peaks deconvoluted from the original spectrum, i.e., 288.6, 286.8, 284.9 and 283.0 eV. These peaks are dedicated to the C(=O) bond, C–C or C–H bond, C–O bond and Si–C bond, respectively [33,42,45,46]. Other than that, the O 1s spectrum displayed two deconvoluted peaks at 532.7 and 530.6 eV, which are dedicated correspondingly to the Si-O-Si and O-H bond [30,31]. Moreover, the three deconvoluted peaks in the Si 2p spectrum at 105.3, 104.1 and 101.9 eV are assigned to the O–Si–O, Si–O and Si–O–C bonds, respectively [45,47,48]. The findings further justified that SiO_2_ and MA had been successfully coated onto the fabric.

### 3.4. Mechanical and Chemical Durability

**Figure 5:**
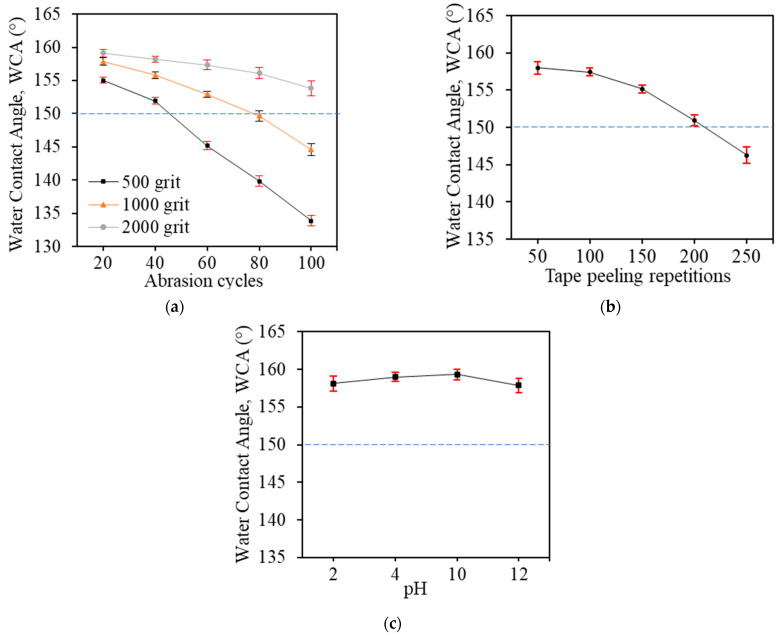
WCA of MA/SiO_2_@cotton filter in (**a**) abrasion tests with various sandpapers, (**b**) tape peelings and (**c**) under different pH conditions.

To evaluate the physical durability of the superhydrophobic filter, sandpaper abrasion and tape peeling were applied to the filter, followed by WCA assessments. The MA/SiO_2_@cotton filter prepared with 0.14 mmol/L of TEOS concentration under 120 °C was selected for the tests. As shown in Figure 5a, the superhydrophobicity of the filter was able to be maintained after 40 cycles of abrasions using SC-500 sandpaper with a WCA of 152°. Beyond that, the MA/SiO_2_@cotton filter lost its superhydrophobicity. This demonstrated that SC-500 sandpaper destructed the surface structure of the filter when the abrasion cycle was >40. The change in surface structure also reduced the superhydrophobicity significantly [32]. However, when employing sandpaper of higher mesh sizes, such as SC-1000 and SC-2000, the modified cotton filter was found to be more stable in maintaining its superhydrophobicity as a higher number of abrasion cycles was required to reduce the hydrophilicity. The WCA results illustrated in Figure 5a confirmed the finding in which the WCA measured for the superhydrophobic filter was 150° after 80 abrasion cycles when using SC-1000 sandpaper and 154° after 100 abrasion cycles for the usage of SC-2000 sandpaper. This is because sandpaper with a smaller mesh size has a higher abrasive capacity and tends to destroy hierarchical structures [32].

To examine the mechanical stability of the prepared SiO_2_/MA@cotton filter against tape peeling, a procedure was executed where an adhesive tape was firmly pressed onto the filter’s surface and then peeled off, considered as one cycle, repeatedly for 250 cycles. After every ten cycles, the tape was replaced with a new piece to maintain the adhesive effect. Figure 5b depicts the changes of WCA every 50 repetitions of the tape peelings. The results showed that the filter coating exhibited excellent stability against tape peeling, with the WCA measured at 151° after 200 repetitions of tape peeling.

The WCA results shown in Figure 5c indicated that the superhydrophobic filter was able to maintain its wetting property after 24-h immersion in solution with a pH ranging from 2 to 12. Furthermore, the WCA measured for the four different pH solutions immersion remained almost unchanged, ranging from 158° to 159°, compared to the initial value of 160°. In short, the MA/SiO_2_@cotton filter demonstrated excellent durability and chemical stability.

### 3.5. Self-Healing Property

**Figure 6:**
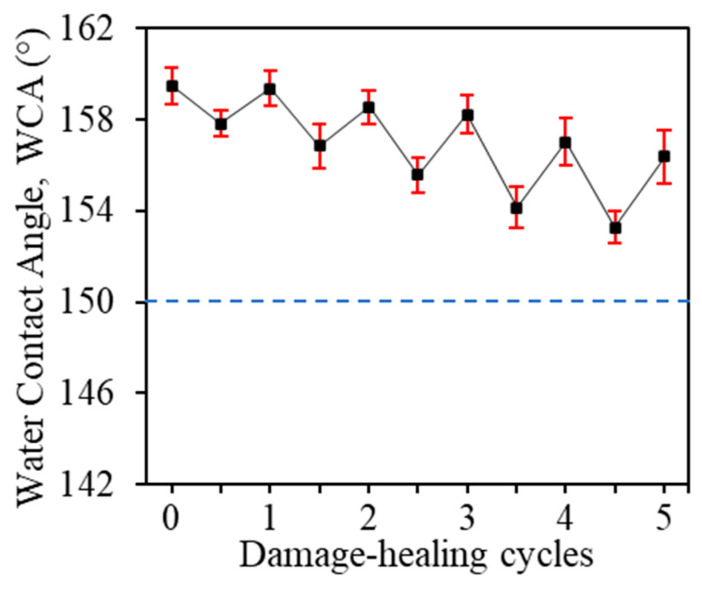
WCA of the superhydrophobic MA/SiO_2_@cotton filter after multiple damage-healing cycles using SC-1000 sandpaper.

The superhydrophobicity of the filter could eventually succumb to mechanical damages, such as abrasion, due to prolonged usage. Interestingly, the ability of the MA/SiO_2_@cotton filter to self-heal after abrasion was discovered. To examine the self-healing ability of the superhydrophobic filter, a set of procedures was developed, which involved subjecting 20 times abrasion to the MA/SiO_2_@cotton filter using SC-1000 sandpaper followed by heating at 80 °C for 2 h. The self-healing property of the filter was examined by the changes in WCA measurements after each set of abrasion and heat treatment procedure was done, as illustrated in Figure 6. The filter was able to be self-recovered to a superhydrophobic state for all five damage-healing cycles, with the final WCA recorded at 156°. When the surface of the superhydrophobic filter was abraded and damaged, the hydrophilic group beneath was exposed to the atmosphere and created a thermodynamically unstable high surface energy region [20]. Due to the difference in surface energy, the low surface energy substances stored in the hierarchical structures will migrate to the damaged area, initiated by external stimuli such as heat, to restore the surface chemistry and complete the self-healing process [15].

In comparison with the changes of WCA after continuous sandpaper abrasions in Section 3.4, where the superhydrophobic filter could only sustain 80 times abrasion done by SC-1000 before losing its superhydrophobic state, the self-healing ability enabled the filter to withstand more abrasion cycles before losing its non-wetting property. This finding further verified the durability of the superhydrophobic MA/SiO_2_@cotton filter against surface wear and tear.

### 3.6. Performance in Oil/Water Separation and Reusability

The prepared MA/SiO_2_@cotton filter can separate oils from oil/water mixture, owing to its unique superhydrophobic and superoleophilic properties. In most of the reported literatures [22,32,49,50], either absorption or filtration was applied for this application. However, this study used the modified cotton fabric for gravity-driven filtration and absorption.

**Figure 7:**
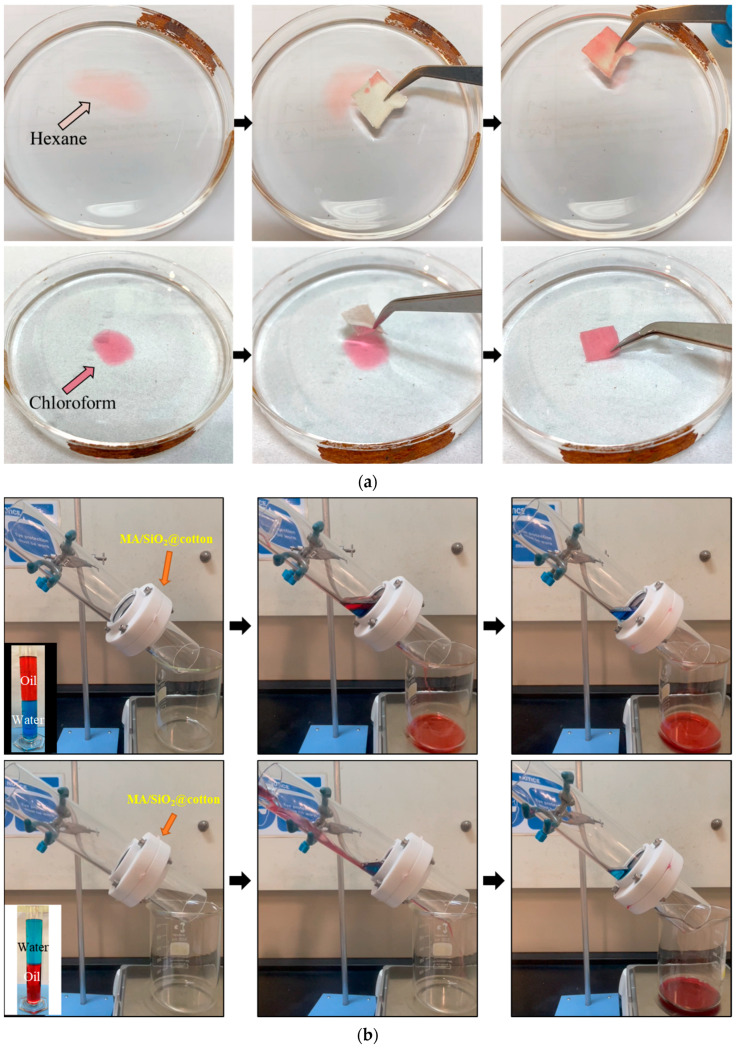

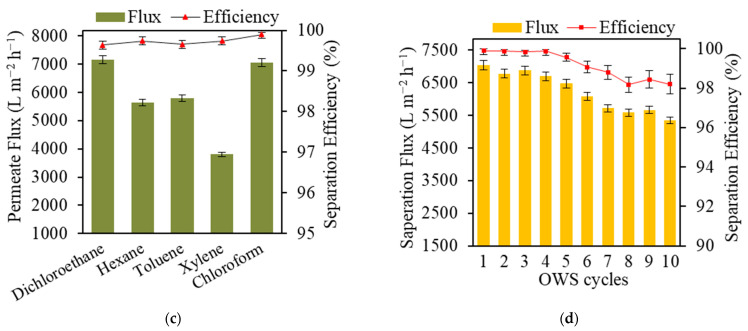
OWS using superhydrophobic MA/SiO_2_@cotton filter in terms of (**a**) oil droplets absorption and (**b**) gravity-driven filtration; The separation flux rate and separation efficiency of the prepared filter in (**c**) separating various types of oils, and (**d**) after ten cycles of separation.

The absorption of oil droplets was carried out, and the results are shown in Figure 7a, where organic oils, such as hexane and chloroform dyed with Oil Red O, were used to imitate the light and heavy oil present in the industrial oily wastewater. By immersing it into the water, the MA/SiO_2_@cotton fabric was observed to selectively absorb the oil droplets either on the water surface or below the water body without any trace of water wetting on the filter, leaving a clean water region behind. The outcome showed that the MA/SiO_2_@cotton fabric has excellent oil absorption properties and water repellency.

OWS is quite a challenging yet important task in removing oils from the oil/water mixture to tackle oil spills or to deal with larger-scale oil/water mixtures, where absorption is not sufficient [51]. Herein, the superoleophilic and superhydrophobic nature of the MA/SiO_2_@cotton fabric was further exploited to separate various oil/water mixtures through filtration under the force of gravity. Figure 7b displays the apparatus setup and the operation of the OWS using hexane and chloroform as examples of light and heavy oil, respectively, in the water mixture. The superhydrophobic cotton fabric was inserted and fixed in the connecting layer of the upper and lower columns of the separation apparatus. Upon pouring the mixture into the upper column, the red-dyed oil penetrated the filter and collected in the beaker as driven by gravity.

Meanwhile, the blue-coloured water remained at the upper column of the setup due to the magnificent water-repellence property of the MA/SiO_2_@cotton fabric surface. Other than that, the experiment was repeated using dichloroethane, xylene and toluene. The separation flux rate and the efficiency of the prepared filter with various oils are depicted in Figure 7c, with the separation flux rate up to 7050 L m^−2^ h^−1^, at an efficiency of up to 99.9%. Furthermore, the MA/SiO_2_@cotton fabric had a permeate flux rate at 5350 L m^−2^ h^−1^ with the separation efficiency as high as 98.2% after 10 OWS cycles (see Figure 7d). The results indicated that the superhydrophobic MA/SiO_2_@cotton filter possesses remarkable performance and reusability in OWS.

The comparison of the separation performance of the MA/SiO_2_@cotton filter with other recently reported works that employed cotton fabric substrate is shown in Table 1. The comparison shows the superiority of our work compared to others because, with the non-hazardous materials used and the simplicity of the fabrication method employed, our superhydrophobic filter exhibits remarkable oil/water separation efficiency and flux rate, with the efficiency of the filter is the highest among all while separating at an extremely high flux rate. Besides, the filter also demonstrated the ability of self-healing and excellent reusability.

## 4. Conclusions

In summary, a fluorine-free and robust superhydrophobic cotton fabric was fabricated using silica sol and myristic acid via a dip coating method followed by a thermal curing process. The optimum TEOS dosage and thermal treatment temperature were determined at 0.14 mmol/L and 120 °C, respectively. By comparing the previous and present findings, the TEOS dosage is independent regardless of the materials used to modify the substrate surface. On the other hand, the optimum curing temperature depends on the material used to modify the surface; hence, increasing the drying temperature can only enhance the superhydrophobicity in a certain manner. Besides, the AFM analysis indicated that the presence of SiO_2_ particles has successfully enhanced the surface roughness of the filter. The prepared MA/SiO_2_@cotton fabric exhibited excellent physical durability against abrasion, tape peeling and chemical stability after 24-h exposure to strong acidic or alkali solution. Furthermore, the MA/SiO_2_@cotton fabric also showed the ability to self-heal, prolonging the service life of the MA/SiO_2_@cotton fabric. Moreover, the superhydrophobic cotton filter also demonstrated remarkable oil/water separation performance in removing light and heavy oil. The filter has a separation efficiency above 98% even after ten oil/water separation cycles with a separation flux rate higher than 5000 L m^−2^ h^−1^. The non-toxic materials used, simple fabrication steps, magnificent durability, self-healing ability, and reusability of the superhydrophobic cotton fabric shall enable a wide application prospect of the prepared filter in the oil/water separation field.

## Figures and Tables

**Table 1:** Comparison of the separation efficiency and flux of superhydrophobic filters in gravity-driven OWS from some recently reported works.

Material(s)	Efficiency *η* (%)	Flux, *J* (L m^−2^ h^−1^)	OWS Cycles, m	*η_m_* (%)	Self-Healing	References
PDMS/ZIF-90/FAS@linen fabric	99.5	1213	10	98	Yes	[25]
Eleostearic acid/SiO_2_@cotton	99.5	3600	15	99.5	No	[52]
SiO2/PAA-b-PS Janus microspheres coated on fabric	99.36	3851	40	98	No	[1]
Rosin acid, SiO_2_ on cotton fabric	98.8	7810	10	96	No	[33]
Single-walled carbon nanotubes (SWCNT) on cotton fabric treated with 1 H,1 H,2 H,2 H-perfluorooctyltriethoxysilane (POTS)	99.2	28,710	20	98.8	No	[53]
Nano-titaniumnitride(TiN), PDMS-SiO_2_ on cotton fabric	98.16	>5500	10	98	Yes	[54]
Myristic acid, SiO_2,_ on cotton fabric	99.9	7050	10	98	Yes	This work

## Data Availability

The data presented in this study are available on request from the corresponding author.
